# Quantitative 18F-FDG PET-CT scan characteristics correlate with tuberculosis treatment response

**DOI:** 10.1186/s13550-020-0591-9

**Published:** 2020-02-10

**Authors:** Stephanus T. Malherbe, Ray Y. Chen, Patrick Dupont, Ilse Kant, Magdalena Kriel, André G. Loxton, Bronwyn Smith, Caroline G. G. Beltran, Susan van Zyl, Shirely McAnda, Charmaine Abrahams, Elizna Maasdorp, Alex Doruyter, Laura E. Via, Clifton E. Barry, David Alland, Stephanie Griffith- Richards, Annare Ellman, Thomas Peppard, John Belisle, Gerard Tromp, Katharina Ronacher, James M. Warwick, Jill Winter, Gerhard Walzl

**Affiliations:** 1 Department of Science and Technology/National Research Foundation, Centre of Excellence for Biomedical Tuberculosis Research and South African Medical Research Council Centre for Tuberculosis Research, Cape Town, South Africa; 2 Division of Molecular Biology and Human Genetics, Faculty of Medicine and Health Sciences, Stellenbosch University, Cape Town, South Africa; 3 Certara, Inc, Princeton, NJ USA; 4 Tuberculosis Research Section, Laboratory of Clinical Infectious Diseases, Division of Intramural Research, National Institute of Allergy and Infectious Diseases, National Institutes of Health, Bethesda, MD USA; 5 Laboratory for Cognitive Neurology, Department of Neurosciences, KU Leuven, Leuven, Belgium; 6 Division of Nuclear Medicine, Department of Medical Imaging and Clinical Oncology, Faculty of Medicine and Health Sciences, Stellenbosch University, Cape Town, South Africa; 7 South African Tuberculosis Bioinformatics Initiative (SATBBI), Faculty of Medicine and Health Sciences, Stellenbosch University, Cape Town, South Africa; 8 Wellcome Centre for Infectious Disease Research in Africa, Institute of Infectious Disease and Molecular Medicine, Faculty of Health Science, University of Cape Town, Cape Town, South Africa; 9 Center for Emerging Pathogens, Department of Medicine, Rutgers-New Jersey Medical School, Rutgers Biomedical and Health Sciences, Newark, NJ USA; 10 Division of Radiodiagnosis, Department of Medical Imaging and Clinical Oncology, Faculty of Medicine and Health Sciences, Stellenbosch University, Cape Town, South Africa; 11 Node for Infection Imaging, Central Analytical Facilities, Stellenbosch University, Cape Town, South Africa; 12 Mycobacteria Research Laboratories, Department of Microbiology, Immunology and Pathology, Colorado State University, Fort Collins, CO USA; 13 Mater Research Institute – The University of Queensland, Translational Research Institute, Brisbane, QLD Australia; 14 Catalysis Foundation for Health, San Ramon, CA USA

**Keywords:** Tuberculosis, 18F-FDG, PET-CT, Tuberculosis treatment response, *Mycobacterium* tuberculosis, Quantitative imaging analysis, Quantified lung analysis

## Abstract

**Background:**

There is a growing interest in the use of F-18 FDG PET-CT to monitor tuberculosis (TB) treatment response. Tuberculosis lung lesions are often complex and diffuse, with dynamic changes during treatment and persisting metabolic activity after apparent clinical cure. This poses a challenge in quantifying scan-based markers of burden of disease and disease activity. We used semi-automated, whole lung quantification of lung lesions to analyse serial FDG PET-CT scans from the Catalysis TB Treatment Response Cohort to identify characteristics that best correlated with clinical and microbiological outcomes.

**Results:**

Quantified scan metrics were already associated with clinical outcomes at diagnosis and 1 month after treatment, with further improved accuracy to differentiate clinical outcomes after standard treatment duration (month 6). A high cavity volume showed the strongest association with a risk of treatment failure (AUC 0.81 to predict failure at diagnosis), while a suboptimal reduction of the total glycolytic activity in lung lesions during treatment had the strongest association with recurrent disease (AUC 0.8 to predict pooled unfavourable outcomes). During the first year after TB treatment lesion burden reduced; but for many patients, there were continued dynamic changes of individual lesions.

**Conclusions:**

Quantification of FDG PET-CT images better characterised TB treatment outcomes than qualitative scan patterns and robustly measured the burden of disease. In future, validated metrics may be used to stratify patients and help evaluate the effectiveness of TB treatment modalities.

## Background

Understanding and accurately measuring the response to tuberculosis (TB) treatment is complex and important. TB is one of the major global killers with an incidence of roughly 10 million and a mortality of roughly 1.6 million people in 2017 [1]. It also commonly affects the most vulnerable communities and often leads to disability associated with post-tuberculosis lung impairment [2–8]. TB is still associated with stigma and ignorance, due to factors such as the infectious nature, resultant chronic wasting, and associations with poverty and other conditions, such as HIV infection and addiction [9].

The protracted treatment of at least 6 months for drug-sensitive pulmonary TB (PTB) increases the burden on health resources and likelihood of non-adherence. In the literature, the reported rate of unfavourable outcomes varies considerably; however, it usually ranges from < 5 to 19% in trials [10–15] to over 20% in national health program conditions [1, 16]. Unfavourable treatment outcomes include failure to convert to sputum culture negativity, treatment default, and disease recurrence, which could be due to either endogenous relapse or exogenous reinfection.

There is considerable effort to improve treatment outcomes, shorten treatment duration, and reduce disability. Nevertheless, testing new antibiotic regimens or immunotherapy options is hampered by the long duration of treatment and follow-up, required because there is no gold standard to determine sterilising cure. Factors contributing to the uncertainty of defining sterilising cure include the persistence of radiological lung lesions [17, 18], clinical symptoms, and *Mycobacterium tuberculosis* (MTB) DNA in sputum [19, 20] after clinical cure. These attributes may persist in spite of sputum culture negativity. Clinical treatment programs, researchers, and investors in new therapies urgently require improved methods to better define TB treatment response. This need has triggered an increasing interest in using 18-F fluorodeoxyglucose positron emission tomography-computed tomography (18-F FDG PET-CT) as a research tool in tuberculosis. Due to its high sensitivity for metabolic activity in infectious lesions, it has shown the potential to be a powerful and possibly cost-effective tool in TB trials, despite the reported lack of specificity in diagnosing active TB in high-incidence areas and its dependence on expensive resources [21, 22].

FDG PET-CT is relatively non-specific for TB, since malignancies and other inflammatory pathology demonstrate similar FDG uptake. Although this limits its use as a TB diagnostic tool in high burden settings [23], its high sensitivity for TB lesions makes it an attractive option to monitor treatment response, once a diagnosis is already established. Firstly, CT is more accurate than traditional chest X-ray for correctly identifying most lesion types associated with TB, especially small nodules, cavities, bronchial thickening, and tree-in-bud lesions [24–26]. Secondly, the addition of PET to CT is reported to further improve sensitivity by identifying small lesions, affected lymph nodes, and helping to distinguish active from inactive lesions [23, 27–29].

Several animal infection models (mice, rabbits, non-human primates) have effectively used FDG PET-CT to shed light on TB progression to disease and response to treatment [29–34]. FDG avidity decreases in lung lesions of MTB-infected animals receiving anti-TB treatment. The reduction in FDG avidity is initially slow (first week of treatment), followed by a sharp decrease in avidity (week 4) after which it stabilises. In untreated animals, FDG intensity shows a variable correlation with MTB load (CFU) and a strong correlation with the lesion size. FDG avidity reduction often precedes reduction of lesion volume and density on CT [29, 31]. The overall reduction in FDG uptake over treatment time correlates with the effectiveness of the bactericidal activity of different treatment options [33].

In mouse models, FDG PET-CT is also able to detect the development of relapse prior to microbiological evidence [30]. Monitoring of the spatial evolution of PTB lesions preceding relapse indicates that there is both progression of existing pre-treatment lesions and the formation of new lesions [35].

Human studies have also shown FDG PET-CT to be promising in monitoring the effect of treatment in pulmonary and extra-pulmonary TB [22, 28, 36–41]. While most of the studies used simple descriptive techniques, two small trials aimed at the treatment of drug-resistant TB, implemented whole lung quantification of PET (using fixed thresholds) and semi-quantified CT reader scores. These studies concluded that quantified PET images were more robust than reader-based CT scores, and both seemed to accurately measure changes in disease burden over time [42, 43].

We recently reported imaging findings in TB patients who underwent FDG PET-CT scans at baseline, during, and after treatment (Catalysis treatment response cohort) [44]. We documented strikingly complex and heterogeneous lesion responses. During treatment, a decrease in size and FDG avidity was noted in most lesions. Unexpectedly, we did however find lesions that appeared metabolically active, with morphology in keeping with active disease in a substantial proportion of PTB patients after standard treatment, including patients with a durable cure and others who later developed recurrent disease.

In this report, we apply quantitative scan assessment by semi-automated whole-lung analysis. We show that these metrics are strongly associated with clinical outcomes, patient factors, and microbiological outcomes. Further, we discuss which identified scan characteristics appear most meaningful for both the interpretation of treatment response and the separation of favourable from unfavourable treatment outcomes. This information is drawn from over 338 scans from 96 patients and points towards the most meaningful metrics in the complex scan profiles of TB treatment response. Some metrics already show prognostic potential at diagnosis, while others that track changes over time become more meaningful at the end of treatment.

## Methods

### Recruitment and study procedures

Participants considered for this report were 99 newly diagnosed, culture-confirmed, pulmonary TB patients who successfully completed follow-up as part of the previously published Catalysis treatment response cohort [44–46]. They were HIV-uninfected adults, recruited at primary health care clinics in the northern regions of Cape Town, South Africa. Patients underwent FDG PET-CT scans at diagnosis (Dx) and at month 1 (M1) and month 6 (M6) of treatment. Fifty patients also had FDG PET-CT scans 1 year after the end of treatment (EOT + 1y). PET images were corrected for attenuation and reconstructed to 4 × 4 × 4 mm voxels using an iterative algorithm. The CT scan parameters were set at 120 kV, 100 mAs, without dose modulation with 1.17 × 1.17 mm pixels, and a 3-mm slice thickness, reconstructed with I31 filter and B31 s con kernel.

**Fig. 1:**
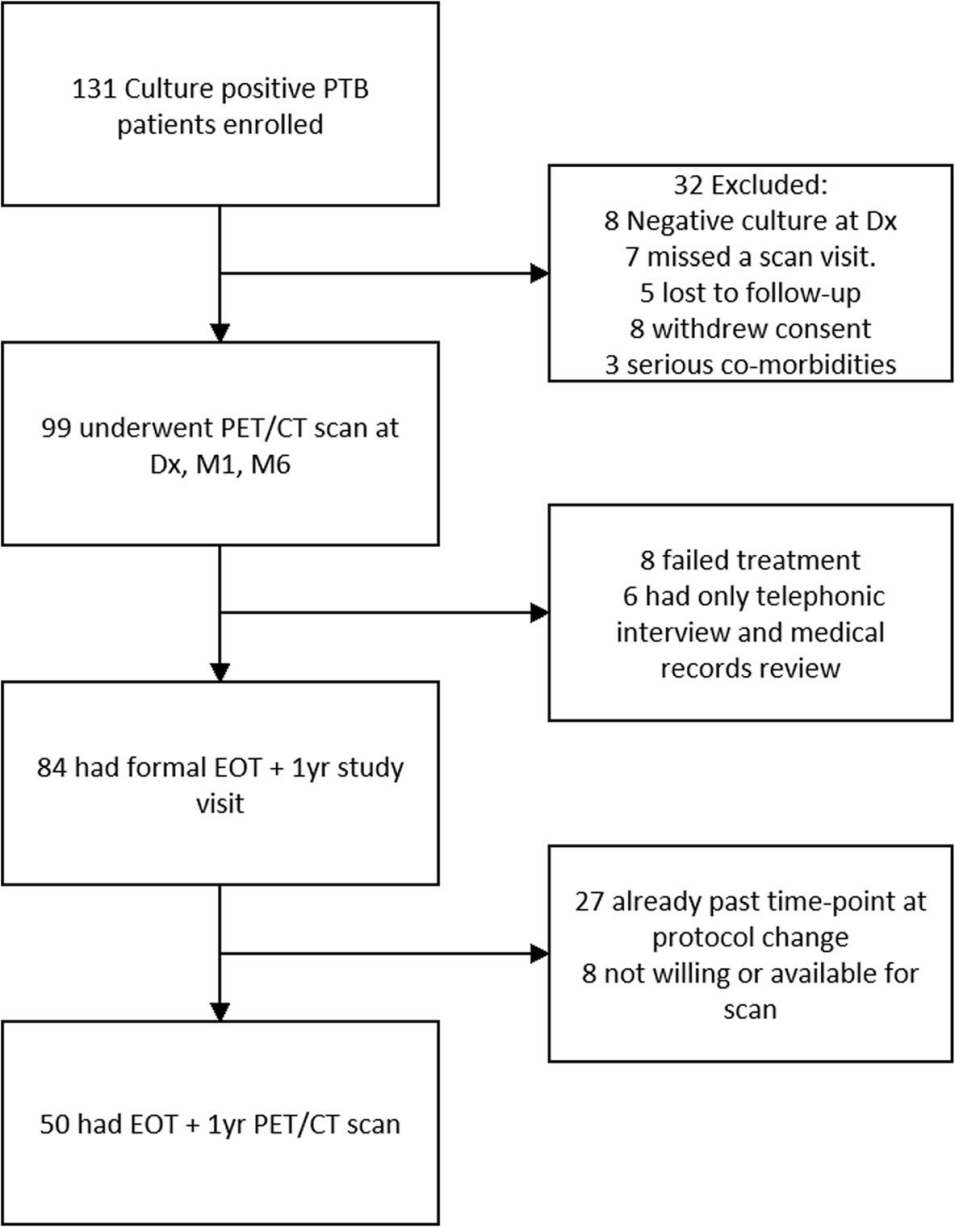
Flow diagram of study design and participants included in analysis

Figure 1 shows a flow diagram of the study design and scan settings as described in Additional file 1: Supplementary note 1.

Clinical samples and information were collected at day 0, week 1, 4, 8, 12, and 24 (month 6) for all participants. Samples included liquid culture with speciation and GeneXpert® MTB/Rif (Xpert) assays on sputum and the analysis of multiple biomarkers in blood and urine.

### Qualitative scan assessment

We previously conducted and reported qualitative scan assessments by comparing each lesion’s intensity at M6 to the intensity at Dx.^46^ Three different response patterns were described: (1) A ‘resolved’ scan response pattern showed no lesion with more than minimally increased FDG intensity when compared to surrounding lung tissue M6. (2) An ‘improved’ pattern indicates that all lesions improved during treatment, but one or more lesion showed residual FDG avidity at M6. (3) A ‘mixed’ response indicated that while some lesions improved, at least one intensified, or a new lesion was present at M6. EOT + 1y scans were compared to M6 scans for qualitative classification.

### Quantitative scan assessment

Based on a previously described methodology [47], we quantified the extent and severity of lung lesions concurrently on PET and CT for all scans (Dx, M1, M6, EOT + 1y). After co-registration of scans across time points using the SPM toolbox [48] in MATLAB (Mathworks Inc.), we created volumes of interest (VOIs) of lungs on the CT component with MRICro [49]. The lung VOIs were adapted by excluding areas affected by misregistration and created to fit all time points. In some cases, we had to create a separate lung VOI for the EOT + 1y scan, due to substantial lung volume changes related to fibrosis or poor inspiratory effort. In addition, we created VOIs which appeared lesion-free on both PET and CT on all time points to represent references for background FDG uptake in the lung.

We segmented the PET component by using a lesion-to-background comparison. To reduce intra- and inter-scan variability, we standardised uptake using patient-specific reference volumes. We assigned a *Z*-score to each voxel based on:

$$ Z=\frac{\mathrm{counts}-{\mu}_{\mathrm{NL}}}{\sigma_{\mathrm{NL}}} $$

in which μ_NL_ and σ_NL_ are the mean and standard deviation of PET counts within the lesion-free lung VOIs for each scan. All voxels exceeding a Z-score of 8 were segmented as FDG-avid [47].

We used a previously reported method of density thresholding to segment lesions on CT [42–47]. These thresholds were as follows: (1) normal density, between − 950 Hounsfield units (HU) and − 500 HU; (2) soft lesions (*V*_soft_), from − 500 to − 300 HU, usually tree-in-bud lesions or nodules, but may also include regular, medium, to large vasculature; (3) medium density lesions (*V*_medium_) from − 300 to − 100 HU, which usually consists of nodular infiltrates, but may also include established lesions in early progression or partial resolution; and (4) hard lesions (*V*_hard_), above − 100HU, are usually due to consolidation, cavity walls, bronchial thickening, or calcified fibrosis. We delineated cavity air volume using a gradient-based region-grow technique, and on the M6 scan measured the thickness of cavity walls at the level of the widest diameter on the transverse view. We measured the wall thickness of enclosed cavities at the level of widest cavity diameter, and the area of maximum wall thickness where there was no confluence with other lesions and structures, or fibrotic changes (examples shown in Additional file 1: Figure S1).

After segmentation, the following PET parameters were quantified: (1) metabolic lesion volume (MLV); (2) the mean *Z*-score in the MLV (*Z*_mean_); and (3) total glycolytic activity index (TGAI): the product of the MLV and mean lesion- to- background intensity index:

(TGAI = MLV × meanlesioncounts/meancountsinnormallung).

In addition, the program also measured the volumes of each abnormal density category on CT, i.e. *V*_soft_, *V*_medium_, *V*_hard_, and total volume with abnormal density > − 500 HU (*V*_total_). We also measured a combined FDG PET-CT metric: MLV_abN_ = the intersection of MLV and area with increased density on CT (≥ − 500 HU).

To create a variable to combine all major contributing factors on PET and CT, we assigned the *Z*_mean_ score to the cavity volume (with no perfusion, thus no FDG uptake). We then added this to the TGAI value to obtain a composite measure of both metabolically active lesions and cavities. The resulting formula was:

$$ \mathrm{TGAIcom}=\mathrm{TGAI}+\left(\mathrm{cavityvolume}\left(\mathrm{ml}\right)\times \mathrm{meanlesioncounts}/\mathrm{meancountsinnormallung}\right). $$

We previously published further detail regarding quantification and evaluation of the described technique [47].

### Statistical testing

We considered a *P* value smaller than 0.05 as significant. We tested the association between categorical and continuous variables with a two-way *T* test for independent samples (Tibco© Staticstica™ V13). For analysis of variance tests with multiple grouping variables (TTN grouping variables), we applied the more conservative Kruskal-Wallis non-parametric test (R version 3.2.2). We calculated *P* values for receiver operating curves in order to test the null hypothesis that the area under the curve equals 0.50 (GraphPad© Prism V8). We performed post hoc analysis to distinguish favourable and pooled unfavourable outcomes by applying thresholds suggested by receiver operating curves. We used the Fisher exact test to determine significant associations between categorical variables (GraphPad© Prism V8). In this descriptive study, we did not correct for multiple testing.

We use the standard terms prognostic and predictive when comparing the association between FDG PET-CT parameters and outcomes. While the ability of a M6 marker to identify failed cases is strictly speaking diagnostic since it applies to the same time point, in practice, culture results are delayed and failed and relapse cases are grouped together when assessing treatment efficacy.

## Results

### Patient demographics and treatment outcome

We recruited 99 PTB patients, of which 95 had drug-sensitive (DS) strains, 2 had isoniazid mono-resistant strains, and 2 multi-drug resistant strains. More details regarding the treatment regimens are provided in Additional file 1: Supplementary note 2.

We based patient clinical outcome classifications on WHO definitions, except we used the more sensitive sputum culture, instead of direct smear microscopy. The outcomes for 3 patients were classified as un-evaluable (UE) due to sputum culture contamination, and they were excluded from the analysis. Of the remaining 96 participants, favourable treatment outcomes include 76 cured cases (achieved and maintained culture conversion). Unfavourable outcomes included 8 failed treatment cases (sputum culture positive at M6), and 12 with recurrent PTB (initially culture converted, but re-diagnosed with active TB within 2 years after treatment completion). One of the 8 failed treatment cases was asymptomatic in spite of a positive sputum culture at M6 and declined to restart treatment and remained symptom-free when assessed a year later. Of the patients with recurrent PTB, 2 were culture confirmed; 5 were confirmed by both Xpert and smear positivity by direct microscopy (acid-fast bacillus positive); 3 were Xpert negative at month 6 but converted back to positive; and 3 remained Xpert positive for more than 6 months and deteriorated clinically. The absence of post-treatment culture complicated the recurrence diagnosis and prevented the distinction between relapse and reinfection.

Outcome was further stratified based on time to culture conversion and treatment adherence. Eighteen patients converted to sputum culture negative within 4 weeks, an additional 39 by week 8, another 22 by week 12, and a further 9 by week 24. Time to culture negativity (TTN) was un-evaluable (UE) for 3 patients due to contaminated cultures. Fourteen patients took fewer than approximately 80% of their treatment dosages during the 6-month period, which is regarded as poor adherence in most clinical trial designs. The failed treatment group included 4 patients with poor treatment adherence and 1 with MDR disease [12, 13]. Further clinical information and demographics of the cohort may be found in our previously published online methods [44].

We performed a fourth scan 1 year after the end of treatment (EOT + 1y) for 50 patients that culture-converted at M6. Eight of these 50 patients were diagnosed with recurrent disease by healthcare providers within 2 years of treatment completion (five before the EOT + 1y scan and three after). The other 42 maintained favourable treatment outcome status.

### Qualitative FDG PET-CT results summary

**Fig. 2:**
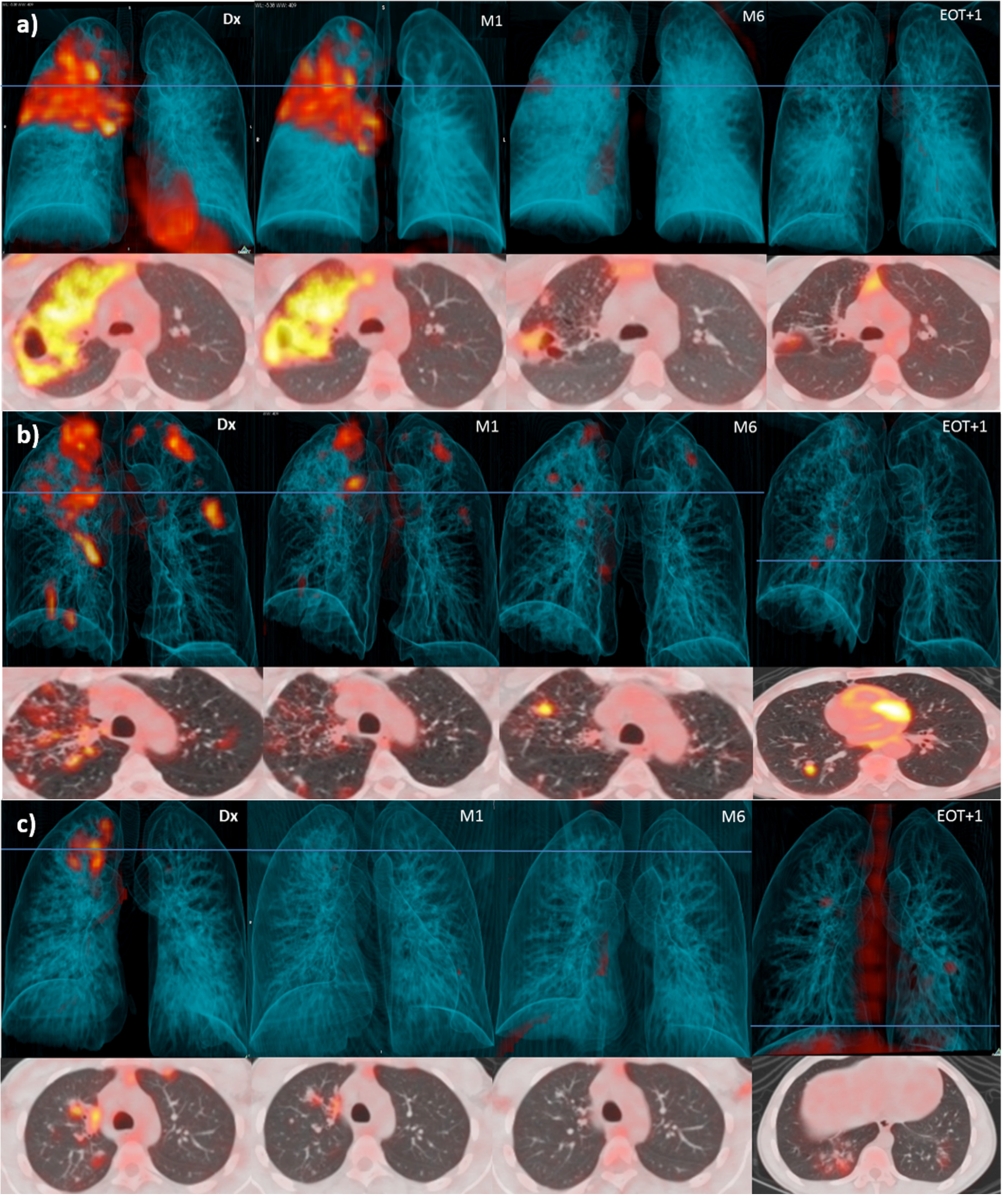
Dx, M1, M6 and EOT + 1y FDG PET-CTs for three representative cases that received 6 months of standard treatment and maintained cure. Three-dimensional anterior and transverse slices at the level of horizontal blue line. **a** Residual cavity with moderate FDG avidity at M6 improves over the next year, leaving nodular infiltrate with mild activity. **b** New nodule with high intensity seen at M6. It resolved at EOT + 1y, but two new nodules have formed. **c** All lesions resolved at M6, but three new areas with small nodular and tree-in-bud infiltrates seen at EOT + 1y

**Fig. 3:**
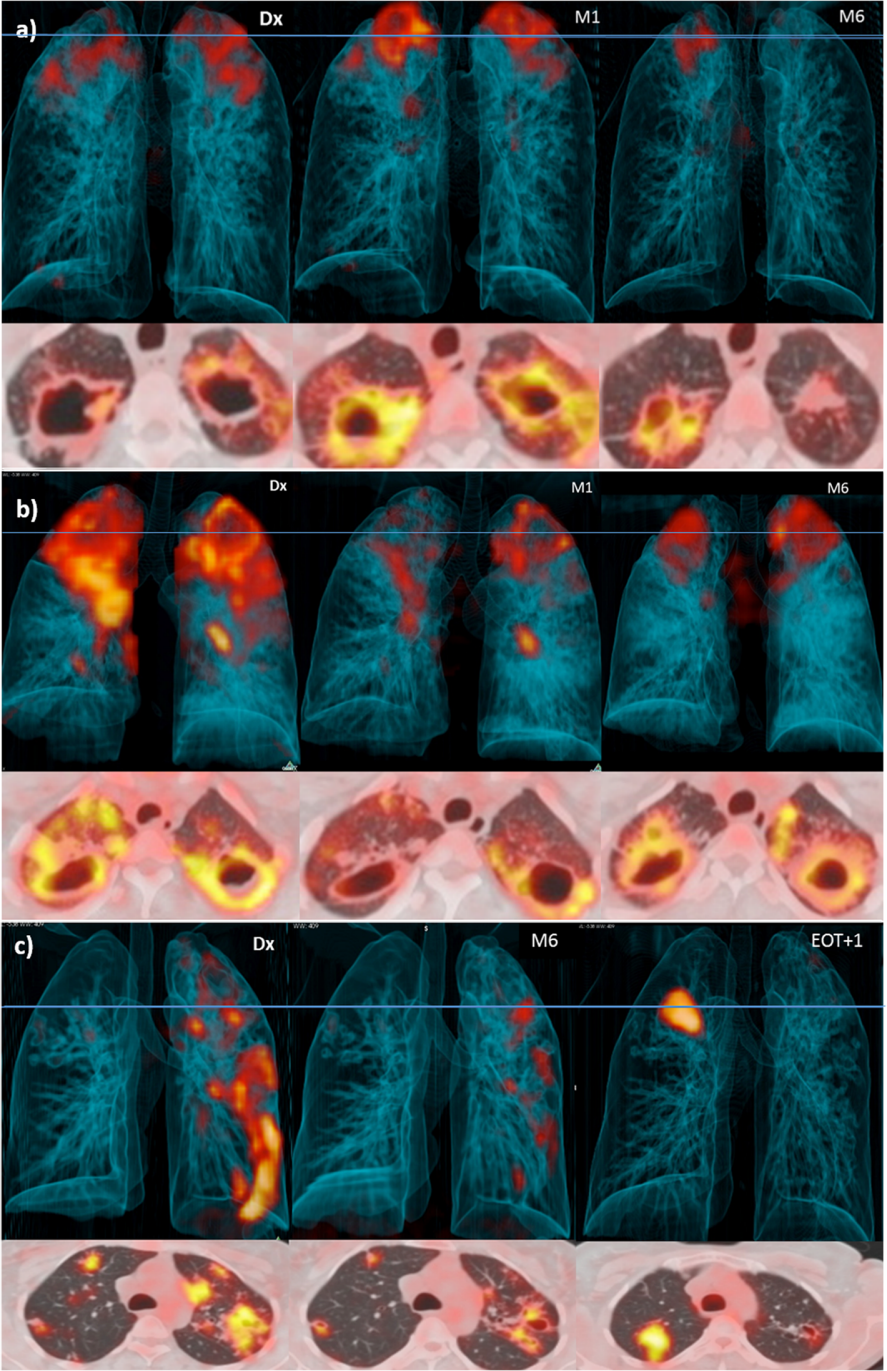
FDG PET-CTs for three representative cases that received 6 months of standard treatment. Three-dimensional anterior and transverse slices at the level of horizontal blue line. **a** Bilateral upper lobe cavitation at Dx, which demonstrate increased intensity at M1. At M6, the left cavity has changed to fibrotic tissue with mild uptake, but the right cavity still has a thick wall and high uptake. **b** Failed treatment case with bilateral upper lobe cavities that retain very high intensity at M6. **c** Case diagnosed with recurrent disease subsequent to EOT + 1y scan. All lesions improved to moderate intensity uptake at M6, but a large new area of patchy consolidation is seen at EOT + 1y

The scans showed ongoing inflammation at the end of treatment in the majority of the patients [44]. For 51 (52%), there was an improved response on the M6 scan (Fig. 2a, Fig. 3a). A mixed response was seen in 34 (34%) patients (Fig. 2b, Fig. 3b), of which 14 had both new and more intense lesion(s), 16 demonstrated only an increase in the intensity of lesion(s), and 4 had only new FDG-avid lesion(s). Only 14 (14%) patients had a resolved pattern on their M6 scan (Fig. 2c).

The morphology associated with the most intense lesion of each mixed and improved M6 scan included CT features suggestive of active PTB, such as cavities (in 26 cases), patchy consolidation (in 22), complex lesions involving consolidation with cavitation (in 16), nodular infiltrates (in 17), enlarged hilar lymph nodes (in 3), and pleural-based infiltrates (in 1). Smaller nodules and tree-in-bud-lesions without calcification tended to resolve during treatment, especially when present in the lower lobes, and even if they were diffuse. Results for each patient are included in Additional file 2: Dataset 1.

### Quantitative FDG PET-CT characteristics in relation to sputum time to culture negativity

**Fig. 4:**
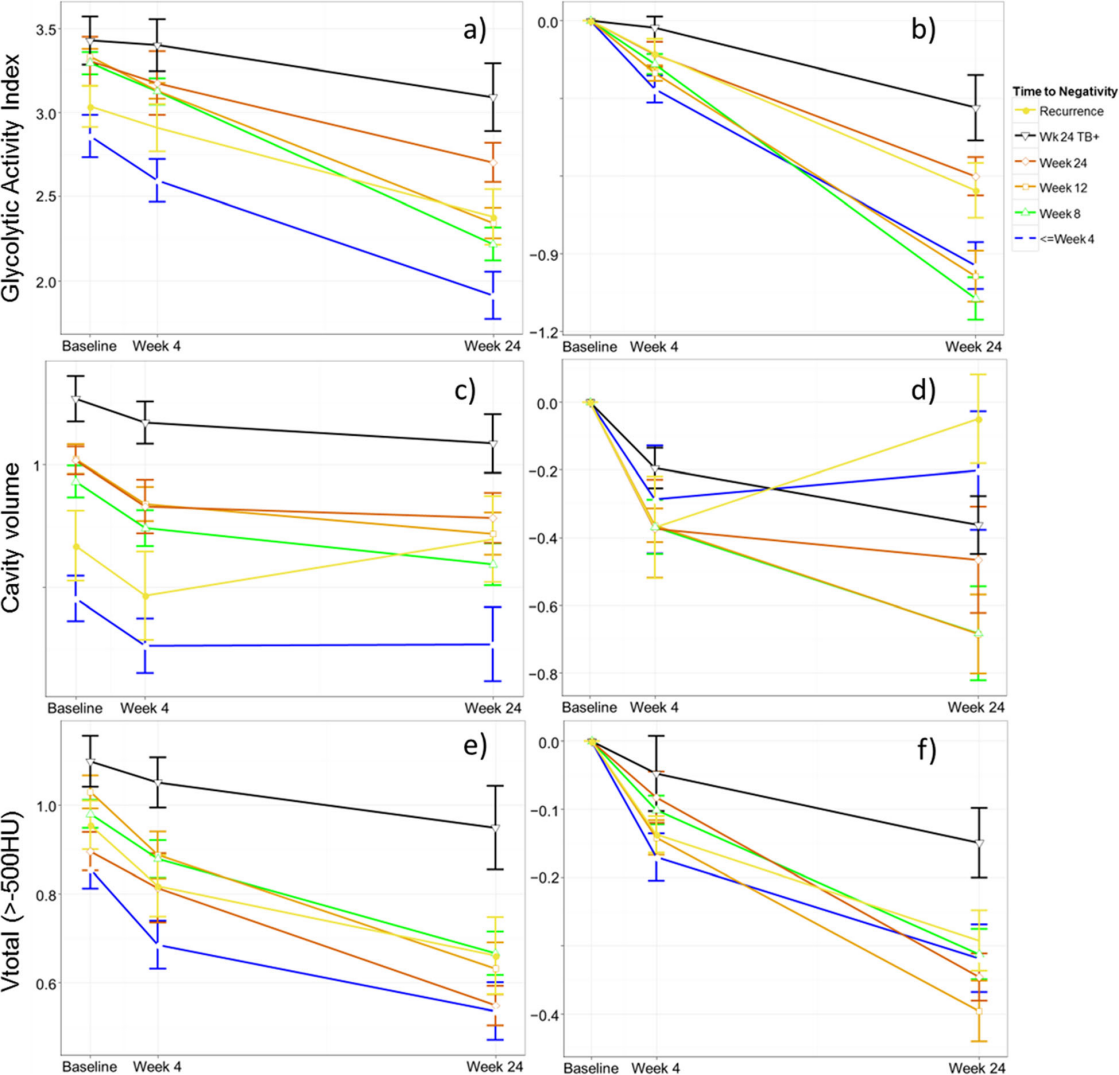
Mean (± SE) log10 transformed values of principal PET and CT parameters over time by time to negativity group and recurrent cases. Total glycolytic activity index in **a** absolute values [ANOVA *P* = 0.002 at Dx and <  0.001 at M1 and M6] and **b** change from baseline [ANOVA *P* = 0.031 at M1 and *P* = 0.002 at M6]. Cavity volume in **c** millilitres [ANOVA *P* < 0.001 at Dx, M1, and M6] and **d** change from baseline [ANOVA *P* = 0.68 at M1 and *P* = 0.165 at M6]. Total high-density CT lesions in **e** percentage of lung volume [ANOVA *P* = 0.013 at Dx, *P* = 0.6 at M1, and *P* = 0.037 at M6] and **f** change from baseline [ANOVA *P* = 0.093 at M1 and *P* = 0.57 M6]

Lesion burden was significantly associated with TTN for the three main independent FDG PET-CT parameters (total cavity volume, TGAI, *V*_total_) at Dx, M1, and M6 (Fig. 4a, c, e). The differentiation between the TTN groups became more pronounced during treatment. Cavity volume showed the largest difference between TTN groups at single time points (*P* < 0.001 at Dx, M1, and M6 —Fig. 4c). Proportional TGAI changes (*P* = 0.031 at M1; *P* = 0.002 at M6 —Fig. 4b) from baseline (delta), were also significantly associated with TTN. Similar trends were noted for cavity volume and *V*_total_ (Fig. 4d, f), but did not meet the threshold for significance. Delayed sputum converters (between months 5 and 6) and failed treatment cases thus showed both a larger burden of disease and a slower rate of reduction in scan metrics. The recurrence group also showed a slower rate of reduction in scan metrics, but did not have a large baseline burden of disease (Fig. 4d).

We also evaluated the individual components of the TGAI (*Z*_mean_, the SUV_max_, MLV —Additional file 1: Figure S3), and high-density lesions on CT (*V*_hard_, *V*_medium_, *V*_soft _—Additional file 1: Figure S4), as well as the intersection of high-density lesions on CT and FDG-avid lesions on PET (MLV_abn_—Additional file 1: Figure S5). We found a significant correlation between TTN groups and single time-point values for indicators of lesion volume (MLV, *V*_hard_, *V*_medium_, *V*_soft_, MLV_abn_), but not for indicators of PET intensity (*Z*_mean_, SUVmax). The proportional change from Dx to M6 in these variables was significantly associated with TTN groups for all these variables (*Z*_mean_, MLV, *V*_hard_, *V*_medium_, *V*_soft_, MLV_abn_) except SUV_max_. None of these variables, however, showed a clear advantage over the main independent FDG PET-CT variables (cavity volume, TGAI, *V*_total_).

### Scan characteristics of failed treatment

As expected from TTN correlation results, cavity volume had the strongest association with treatment failure, with an area under the curve (AUC) of 0.81 (*P* = 0.006) at Dx, 0.83 (*P* = 0.005) at M1, and 0.87 (*P* = 0.004) at M6 (Additional file 1: Figure S6). Apart from cavity volume, other metrics reflecting lesion extent (*V*_total_, MLV, MLV_abn_) also showed promise to differentiate failed cases from cured at Dx, M1, and M6, while parameters reflecting the intensity of FDG uptake (SUVmax, *Z*_mean_) did not show prognostic value at baseline, but only at M6. A summary of AUC’s for various scan parameters to differentiate failed treatment cases is in Additional file 1: Table S1. Of note, the one asymptomatic, failed treatment case (participant identification number 43) had quantified values in keeping with a good response to treatment.

In addition to cavity volume, M6 cavity wall thickness was also associated with treatment failure. In most cured cases, M6 cavity wall thickness ranged from 0 (no cavity) to 3 mm. M6 cavity wall thickness in failed cases was significantly greater than cured cases’ (Student’s *T* test for independent samples; *P* <  0.001) and ranged from 2.5 to 8 mm. At end of treatment, recurrent cases were not significantly different from other cured cases; their M6 cavity wall thickness ranged from 0 to 4 mm.

Treatment outcome was also associated with the qualitative scan response pattern (Fisher’s exact test; *P* <  0.01) and showed high sensitivity, in that a mixed response was found in all failed patients at M6. However, neither a mixed response nor a high maximum lesion intensity was specific for an unfavourable outcome, and 21 (28%) of cured patients had a mixed response, while 55 (72%) still had M6 lesions with moderate to very high intensity. This was similar to the intensity range seen in some untreated cases at diagnosis.

### Scan characteristics of recurrence

The 12 patients diagnosed with recurrent disease within 2 years after treatment had a similar sputum culture conversion rate to cured cases (median TTN 8 weeks) and did not show a comparatively large lesion burden at Dx (Fig. 4). Nevertheless, irrespective of TTN, during treatment they exhibited a relatively slow rate of reduction in TGAI, cavity volume, and to a lesser extent *V*_total_ (Fig. 4b, d, f).

At M1, there was a trend for the recurrent disease group to have a smaller reduction in TGAI and TGAI_com_ burden. At M6, the difference between the groups was significant (*P* = 0.003). No other parameters were significantly different between cured and recurrent disease groups.

Patients who reported previous PTB episode(s) tended to have a higher TGAI burden at Dx and showed significantly less TGAI reduction on treatment (*P* = 0.003, Additional file 1: Figure S7). They also showed less reduction in cavity volume, but no clear difference in abnormal CT density (*V*_total_). See Additional file 1: Table S2 for additional summary statistics on previous TB.

### Scan characteristics of pooled unfavourable outcomes

**Fig. 5:**
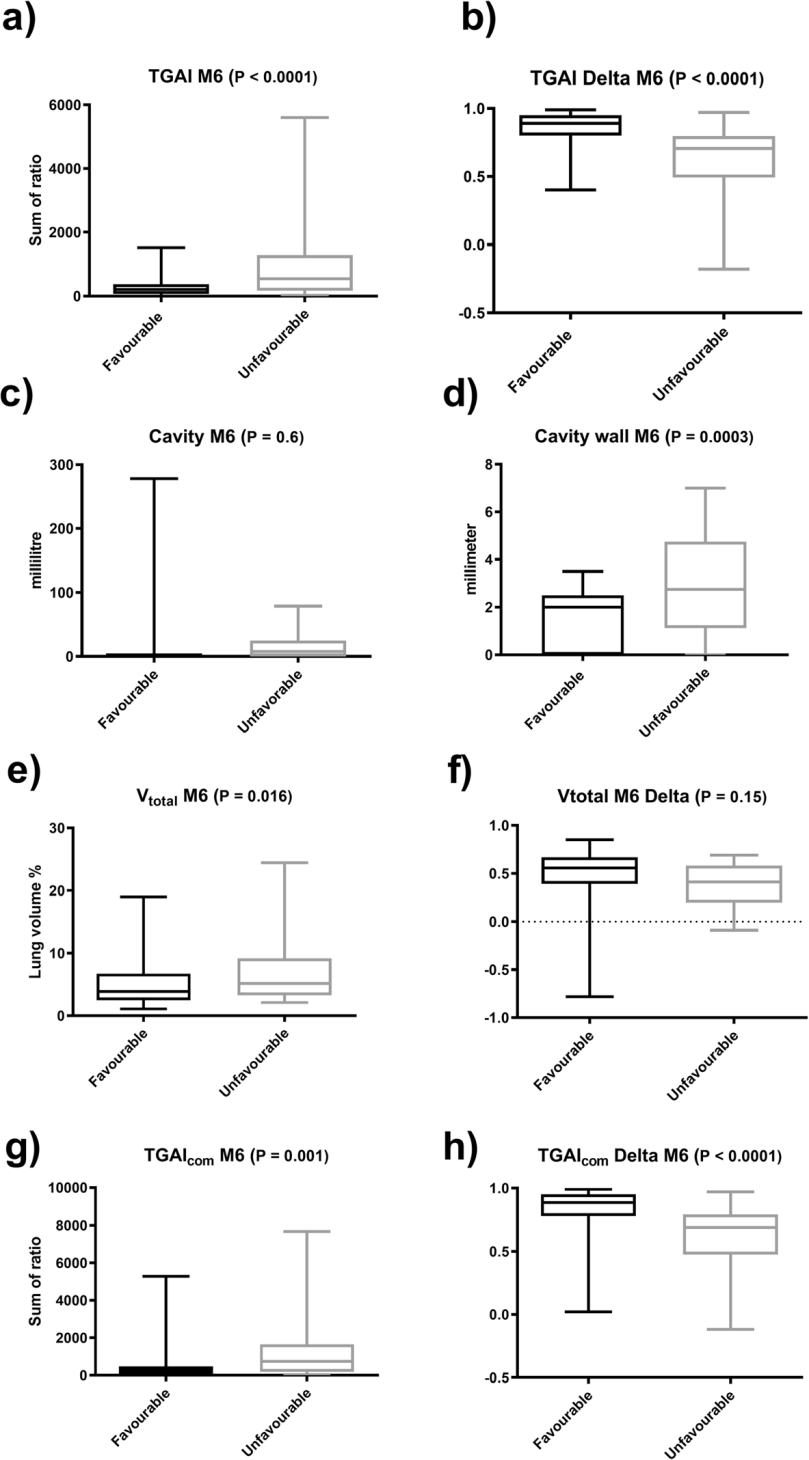
Box and whisker plots showing median, 25th and 75th percentile (box), and range (whiskers) of scan metrics at M6, grouped by favourable and unfavourable treatment outcome. *P* values calculated by Student’s *T* test for independent samples. **a** Total glycolytic activity index (TGAI). **b** Change in TGAI from Dx to M6. **c** Total cavity volume. **d** Cavity wall thickness. **e** Total abnormal density lung volume. **f** Change in total abnormal density lung volume from Dx to M6. **g** TGAI_com_ at M6. **h** Change in TGAI_com_ from Dx to M6

We pooled patients with unfavourable outcomes (failed and recurrent treatment) and analysed the most promising scan parameters’ distribution per groups, combined with receiver operating curve analysis to determine the most informed thresholds. A failure to reduce TGAI by less than 80% from Dx to M6 was the scan characteristic most associated with unfavourable outcomes and carried an almost sevenfold risk. Table 1 shows indicators of M6 scan parameter associations with unfavourable outcomes and the suggested cut-offs, and Fig. 5 compares the distribution of scan metrics for outcome groups.

**Table 1:** Summary of contingency table statistics for scan parameters

										No. that met criteria (*n* = 96)		
Parameter	*P* value	Relative risk	95% CI	Sens	Spec	PPV	NPV	AUC	Criteria	Cured	Fail	Recur
TGAI change M6	< 0.0001	6.97	2.53–19.22	0.80	0.75	0.46	0.94	0.80	< 80%	19	7	9
TGAIcom change M6	< 0.0001	6.67	2.42–18.40	0.80	0.74	0.44	0.93	0.80	80%	20	7	9
Cavity M6	< 0.001	4.36	2.09–9.12	0.55	0.86	0.52	0.88	0.65	> 7 ml	10	7	4
Cav change M6	< 0.01	4.30	1.91–9.64	0.65	0.79	0.45	0.90	0.68	< 60%	16	6	7
M6 TGAI_com_	< 0.001	4.05	1.98–8.32	0.50	0.88	0.52	0.87	0.68	> 1000	9	6	4
TGAI M6	< 0.001	4.05	1.98–8.33	0.50	0.88	0.53	0.87	0.69	> 600	9	6	4
Cavwall M6	0.00	3.95	1.90–7.90	0.50	0.88	0.53	0.87	0.70	≥ 3 mm	9	6	4
Mixed response M6	0.02	2.86	1.30–6.31	0.60	0.72	0.36	0.87	N/A	Intensified	21	8	4
*V*_total_ M6	0.05	2.20	1.03–4.56	0.45	0.77	0.35	0.84	0.64	> 7%	17	6	3
SUVmax M6	0.19	1.82	0.85–3.85	0.50	0.68	0.29	0.84	0.60	> 4	24	6	4
Cav change M1	0.01	2.80	1.33–5.92	0.50	0.80	0.40	0.86	0.52	< 33%	15	5	5
Cavity M1	0.04	2.50	1.18–5.32	0.35	0.87	0.41	0.84	0.57	> 20 mm^3^	10	6	1
TGAI change M1	0.07	2.24	1.05–4.78	0.40	0.82	0.36	0.84	0.67	< 5%	14	4	4
TGAI_com_ change M1	0.16	1.80	0.83–3.89	0.40	0.76	0.31	0.83	0.66	15%	18	3	5
*V*_total_ change M6	0.01	2.89	1.39–5.77	0.40	0.87	0.44	0.85	0.66	< 50%	10	5	3
Cavity Dx	0.18	1.72	0.79–3.71	0.45	0.72	0.29	0.83	0.53	> 16.5 ml	22	6	3

Ranked according to relative risk of unfavourable outcome. Fisher exact test was performed to determine significance. Sens (sensitivity), spec (specificity), *PPV* (positive predictive value), NPV negative predictive value. Change (change from baseline), intensified (at least one intensified or new lesion), month 6 (M6), percentage change from baseline to M6 (change), total glycolytic activity index (TGAI), composite TGAI (TGAI_com_), cavity wall thickness (Cavwall), total abnormal density volume (*V*_total_)

A total M6 cavity volume greater than 7 ml and a M6 TGAI of greater than 600 (equivalent to a SUV-based total glycolytic activity of roughly 200 if calculated using SUV) also carried a fourfold increased risk of an unfavourable outcome. Cavity volume was slightly more sensitive and TGAI more specific in predicting unfavourable outcomes. Combining the variables did not improve prognostic accuracy, either when merged into a single variable or when used in Boolean selection. TGAI_com_ performed very similarly to TGAI. We also tested whether either M6 cavity volume > 7 ml or TGAI > 600 put a patient in the high-risk group, but this generated the same sensitivity but lower specificity than single variables.

Quantitative parameters at M6 out-performed lesion-based qualitative measurements. A mixed response pattern (either new or intensified lesions) at M6 was associated with a 2.86 times increased risk of unfavourable outcome, which was comparable to the prognostic potential of the quantitative parameters at M1. At M1, both a cavity volume greater than 20 ml and a less than 33% cavity volume reduction from Dx were significantly associated with unfavourable outcome, showing a 2.5- and 2.8-times increased risk of unfavourable outcome respectively (*P* = 0.04 and *P* = 0.01 respectively). Total TGAI values at Dx and M1 did not perform well as a predictor of pooled unfavourable outcomes, but a trend (*P* = 0.07) suggests an increased risk of unfavourable outcome when there is less than 5% reduction in TGAI (from Dx to M1).

### Scan results: EOT + 1y

**Fig. 6:**
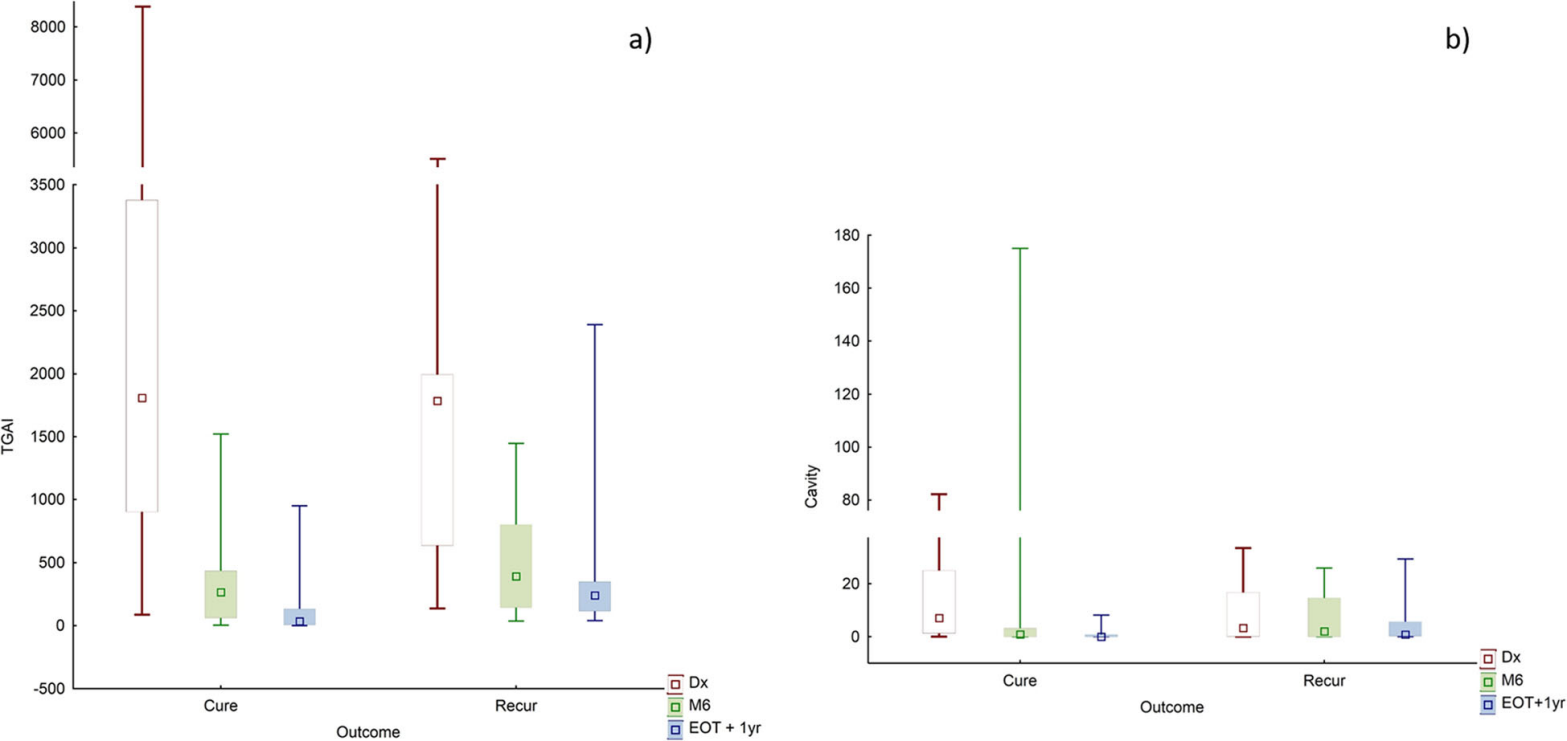
Box and whisker plots showing median, quartiles, and range, grouped in cured and recurrent patients. Y-axis truncated. **a** TGAI at Dx, M6, and EOT + 1y. **b** Total cavity volume at Dx, M6, and EOT + 1y

Most residual lesions were smaller and less intense 1 year after the end of treatment. Metabolic lesion volume decreased to an average of only 2.03% of lung volume at EOT + 1y, compared to 4.21% at M6 in the same patients. Mean total cavity volume also decreased from 7.6 ml at M6 to 2 ml at EOT + 1y. Abnormal CT density showed less reduction during treatment than other parameters after treatment, and the mean *V*_total_ at EOT + 1y was 4.53%, compared to 5.7% at M6. Figure 6 shows the distribution of TGAI and cavity volume across time points.

Remarkably, only 32% of EOT + 1y scans were completely resolved. The remaining 68% had FDG-avid residual lesions, of which half had improvement of all lesions compared to M6 (Fig. 2a), and the other half had a mixed lesion response compared to the M6 scan (Fig. 2b, c, Fig. 3c). Morphology of new FDG-avid lesions at EOT + 1y included nodular infiltrates (found in 4 cases), hilar lymph nodes (in 1), cavitation (in 2), consolidation (in 2), or lesions with combined morphology (in 3). There was no association between the development of new lesions during Dx-M6 and during M6-EOT + 1y. Morphology of residual M6 lesions showing similar or more intense FDG uptake at EOT + 1y included consolidation (2), cavitation (4), and nodules (2). All three patients who developed recurrent PTB after EOT + 1y had mixed scan outcomes at this time point (Fig. 2c), while none with resolved EOT + 1y scans were diagnosed with recurrence. Figure 2 and Fig. 3 show examples of dynamic lesion progression and resolution during and after treatment.

We found no significant association between M6 and EOT + 1y for TGAI or cavity volume (Additional file 1: Figure S8a and S8b). However, we found a moderate correlation between the time points for *V*_total_ and SUVmax (Additional file 1: Figure S8c and S8d).

## Discussion

### Summary of main findings

Quantification of the FDG PET-CT images provides metrics that show stronger association with clinical outcomes compared to qualitative scan patterns. Qualitative scan response patterns are more challenging to interpret, due to varying responses of individual lesions and incomplete resolution of inflammation during treatment. The most promising quantitative marker (TGAI not reducing by > 80% from Dx to M6) carried a 6.97 relative risk of unfavourable outcome, compared to 2.86 if a mixed response pattern was observed.

Various scan metrics measured in this study showed prognostic potential at Dx and M1 and stronger associations with unfavourable outcomes by M6. A high cavity volume showed the strongest association with a risk of treatment failure, while a suboptimal reduction of the total glycolytic activity throughout the lung had the strongest association with recurrent disease. Both of these variables also correlated with time to culture negativity. This suggests a correlation between the quantified lesion burden and the MTB load that is clearer when using quantitative rather than qualitative analysis.

The volume of high-density lesions on CT (*V*_total_) also shows a strong association with TTN and failed treatment, even at early time points (Dx and M1). Unlike TGAI, however, *V*_total_ shows no association with recurrent disease and subsequently, pooled unfavourable outcomes. This is likely due to residual scarring and fibrotic changes and the related residual abnormal density lesions on CT after treatment. Values indicating FDG uptake intensity alone (SUVmax and *Z*_mean_) do not show association early in treatment. However, the proportional intensity changes are associated with TTN and outcome, though not as strongly as TGAI, which combines information from intensity and volume. Combined PET and CT parameters perform similarly to their underlying components, but they do not appear to be clearly superior to individual variables.

Quantification of EOT + 1y scans confirms our previous observations that there is a tendency for all parameters to decrease after treatment, but that a lack of complete resolution is still common and new or intensifying lesions are often seen. Dynamic changes after treatment are common for PET parameters resulting in a poor correlation between M6 and EOT + 1y measurements, compared to CT lesions which appear to be more persistent after treatment.

We found notable differences between failed and recurrent treatment cases. Failed treatment is associated with extensive lung lesions at baseline and large cavities with thick walls at M6, as well as poor adherence. On treatment, a reduction in the FDG avidity and thickness of cavity walls is usually also associated with a reduction in cavity volume. Interestingly, it is relatively common for cavities to show a reduction in both FDG avidity and wall thickness (thus appearing inactive), but to show an increase in size after M1. This may reflect a loss of structural wall strength and progress towards the formation of bullae (Additional file 1: Figure S1b). Recurrent cases display a comparatively low lesion burden at baseline, average adherence, and time to sputum culture negativity, but insufficient reduction in lesion burden during treatment. We also found insufficient reduction in lesion burden in patients with a history of previous PTB episodes.

### Comparison with previous literature

The catalysis treatment response cohort is the largest prospective study conducted on the use of FDG PET-CT in human patients with PTB and the first report on the fate of residual FDG PET-CT lesions after PTB treatment [45]. In this report, we found that a quantitative analysis of scan characteristics shows a stronger association with outcomes than a qualitative analysis of these same characteristics. In related publications, these quantitative metrics also correlate well with host biomarkers, namely gene expression signatures [46], and urinary concentration of the recently discovered metabolite, seryl-leucine core 1 O-glycosylated peptide [50]. The potential of quantitative FDG PET-CT variables to identify patients with a low risk of treatment failure was also analysed in combination with other patient variables and is currently being tested in the PredictTB trial [51].

Our quantitative findings correspond well with previous reports on animal models [29–31, 52] and validate findings from two studies in drug-resistant TB cases, which also show that the quantified inflammation burden, as measured by FDG, corresponds with the effectiveness of treatment [41, 43]. Our results are consistent with those of previous reports in humans in which cavitary disease is associated with an unfavourable outcome [11, 53, 54]. The persistence of density changes in the lungs is also in keeping with reports of the high incidence of post-tuberculosis lung impairment [6, 8]. We found no published reports in human tuberculosis that compare as many quantified parameters in either PET or CT scans for a sample size this large or suggest cut-off values that may be used for direct comparison in future studies.

### Study limitations

Although this is the largest prospective cohort of FDG PET-CT in TB treatment response, it is still a limited sample size with a small number of unfavourable outcomes, and we did not have sufficient data to differentiate relapse from recurrence. On account of these limitations, we did not perform multivariable logistic regression analysis, due to both the risk of false-positive findings from overfitting a model and false-negative findings due to confounding unfavourable outcomes by reinfection cases. In the absence of a ground truth, we also cannot exclude the possibility that other analysis methods could improve on the prognostic ability of scan characteristics or that different PET-CT scanner models, acquisition, and reconstruction protocols will affect the results. With regard to EOT + 1y scans, the variation in timing between the scan and the recurrent disease diagnosis limited the conclusions we can draw from the data. The study design excluded HIV-infected participants to ensure a more homogenous group for biomarker discovery. As such, the suitability of the variables and cut-offs will have to be re-evaluated in this important subset of TB patients. We did not perform pharmacokinetic studies.

### Implications

At month 6, the best indicator of unfavourable outcomes (treatment failure and recurrences) shows 80% sensitivity and 75% specificity, which is modest for a diagnostic test but far superior to currently used predictive biomarkers for poor treatment outcomes, such as month 2 sputum culture conversion or AFB smear conversion. The thresholds defined in this report require further validation before direct application to clinical practice, but the associations with quantified patient and microbiological data suggest that most prominent FDG PET-CT scan characteristics can be used as part of risk stratification and treatment response monitoring in therapeutic trials. In combination with clinical evidence, it may also assist treatment decisions in complicated clinical cases, such as treatment of drug-resistant TB and evaluation of adverse reactions to medication. Quantification of central trends in lesion burden provides continuous variables that allow multiple options for statistical analysis. This approach holds promise for improving the accuracy of clinical reporting.

### Future research

Quantification methods should be further improved to be less operator-dependent, more user-friendly and widely available to both researchers and clinicians. Suggested thresholds should be validated and tested in shortened TB regimens. Further translational research may implement FDG PET-CT scan characteristics to explore complex interactions between host, MTB, and anti-tuberculous drugs, to help develop improved regimens, host-directed therapy, and diagnostic tests.

## Conclusions

Quantification of FDG PET-CT images better characterised TB treatment outcomes than qualitative scan patterns and robustly measured the burden of disease. This approach requires validation in future studies.

## Supplementary information

**Additional file 1:** Supplementary notes 1 and 2, Supplementary Tables S1 and S2, and Supplementary Figures S1–S8

**Additional file 2:** Supplementary dataset 1

## Data Availability

Datasets are added as Additional file 2: Dataset 1, and more detailed data are publically available on request. Please visit https://c-path.org/programs/cptr/cptr-tools/databases/cptr-cdc-study-data/

## Abbreviations

CT: X-ray computed tomography
Dx: Diagnosis
FDG: 2-Deoxy-2-(Fluorine-18)fluoro-D-glucose
HU: Hounsfield units
M1: Month 1
M6: Month 6
MLV: Volume with high intensity (> *Z*-score 8)
MLV_abn_: Volume with high density (> − 500HU) and high intensity (> *Z*-score 8)
PET: Positron emission tomography
PTB: Pulmonary tuberculosis
SUV: Standard uptake value
TGA: Total glycolytic activity (SUV_mean_ × lesion volume)
TGAI: Total glycolytic activity index (mean lesion-to-background × lesion volume)
TGAI_com_: Composite total glycolytic activity
*V*_hard_: Hard lesions volume (> − 100 HU)
*V*_low_: Hypodense lesions (< − 950 HU)
*V*_medium_: Medium lesion volume (− 300 HU to − 100 HU)
VOI: Volume of interest
*V*_soft_: Soft lesion volume (− 500 HU to – 300 HU)
*Z*: Z-score/standardised score
*Z*_mean_: Mean standardised intensity
