# Supplementary material for: Quantitative 18F-FDG PET-CT scan characteristics correlate with tuberculosis treatment response
Source: EJNMMI Res. 2020 Feb 10;10:8. doi: 10.1186/s13550-020-0591-9 (PMC7010890; doi:10.1186/s13550-020-0591-9)
Supplement: Supplementary file 1 — Additional file 1: Supplementary notes 1 and 2, Supplementary Tables S1 and S2, and Supplementary Figures S1–S8 [file 13550_2020_591_MOESM1_ESM.docx]

# Supplementary note 1: scan settings

Scans were performed at Panorama Mediclinic or Tygerberg Hospital, using a Siemens Biograph or Philips Gemini scanner respectively, both of which were equipped with a 16 slice CT scanner. Patients fasted for at least 6 hours before FDG administration, while being encouraged to drink plenty of water. According to body weight 185-259MBq of ^18^F -FDG was administered intravenously 60 min before scan. Scans at baseline were performed from the base of skull to the upper thigh and from the neck to the upper abdomen (whole lung) at M1, M6 and EOT + 1y. PET images were reconstructed to 4x4x4 mm voxels using an iterative algorithm including time-of-flight information and corrections for random events, scatter, deadtime, attenuation and decay. The CT scan parameters were set at 120kV, 100mAs, rotation 0.5 s, pitch 0.813, collimation 16 × 1.5, without dose modulation with 1.17x1.17 mm pixels and a 3 mm slice thickness; reconstructed with I31 filter and B31 s con kernel. A one-week window was allowed for the Dx and M1 scans, while a 4-week window was allowed for M6 and EOT + 1y year scans.

# Supplementary note 2: Treatment regimens

Seventy-two patients received a standard treatment regimen, consisting of a two months intensive phase with daily fixed-dose combination tablets (HRZE) consisting of isoniazid (INH), rifampicin (RIF), ethambutol (EMB) and pyrazinamide (PZA) followed by a 4 month continuation phase of daily INH and RIF (currently the standard of care according to the WHO guidelines). ^51^ Twenty-three patients received an extended regimen: 3 months initiation phase of HRZE, with additional streptomycin in the first month (19) or without (4), and a 5 months continuation phase. In 22 patients, the indication for the extended regimen was previous episode(s) of PTB and for the remaining patient, delayed smear conversion. The local guidelines were amended during the study period to standard 6 months of HRZE treatment for all drug-susceptible cases, resulting in seventeen patients with previous episode(s) not receiving extended treatment.

All patients whose treatment regimen was longer than 6 months maintained an unchanged sputum culture status between M6 and EOT. Note that M6 scan time-points occurred after 6 months of treatment (consistent with standard length of treatment., while EOT + 1y scans happened 1 year after the date that treatment were completed.

Two patients had INH mono-resistance and received 6 months of HRZE with added ofloxacin and 12 months of HRZE respectively. Two patients with MDR received individualised regimens, containing kanamycin and terizidone among others.

# Supplementary Table 1

*Area under the curve for scan parameters to identify failed cases at different time-points.*

|  | **Dx** | **M1** | **M6** |
| --- | --- | --- | --- |
| **TGAI** | 0.67 | 0.75 | 0.84 |
| **MLV** | 0.7 | 0.76 | 0.84 |
| **Zmean** | 0.37 | 0.43 | 0.79 |
| **SUVmax** | 0.45 | 0.51 | 0.7 |
| **Cavity vol** | 0.81 | 0.83 | 0.87 |
| **Cavity wall** | n/a | n/a | 0.87 |
| **Vsoft** | 0.69 | 0.77 | 0.72 |
| **Vmed** | 0.71 | 0.78 | 0.86 |
| **Vhard** | 0.71 | 0.7 | 0.85 |
| **Vtot** | 0.71 | 0.75 | 0.8 |
| **MLVabn** | 0.68 | 0.77 | 0.86 |
| **TGAIcom** | 0.7 | 0.76 | 0.87 |

Supplementary Table 2 T-test results testing for difference in M6 PET/CT parameters and history of previous PTB.

|  | 1st episode Mean | Previous PTB mean | T-test P- value | Levene P-Value | 1st episode (n =) | Previous PTB (n =) |
| --- | --- | --- | --- | --- | --- | --- |
| TGAI Dx | 2279.98 | 3054.43 | 0.083 | 0.033 | 68 | 31 |
| TGAI M6 | 302.07 | 891.25 | **0.001** | **<0.001** | 68 | 31 |
| TGAI Change (Dx–M6) | 0.84 | 0.70 | **0.003** | **0.003** | 68 | 31 |
| Cavity1 | 18.04 | 22.41 | 0.526 | 0.798 | 68 | 31 |
| Cavity3 | 11.01 | 9.96 | 0.893 | 0.479 | 68 | 31 |
| Cav Change (Dx–M6) | 0.65 | 0.45 | 0.211 | 0.209 | 64 | 31 |
| TGAIcom (Dx–M6) | 472.43 | 1079.44 | **0.016** | **0.005** | 68 | 31 |
| TGAIcom Change (Dx–M6) | 0.82 | 0.71 | **0.025** | 0.058 | 68 | 31 |
| Vtotal Dx | 5.41 | 5.95 | 0.428 | 0.413 | 68 | 31 |
| Vtotal M6 | 1.83 | 2.38 | 0.266 | 0.725 | 68 | 31 |

# Supplementary Figures


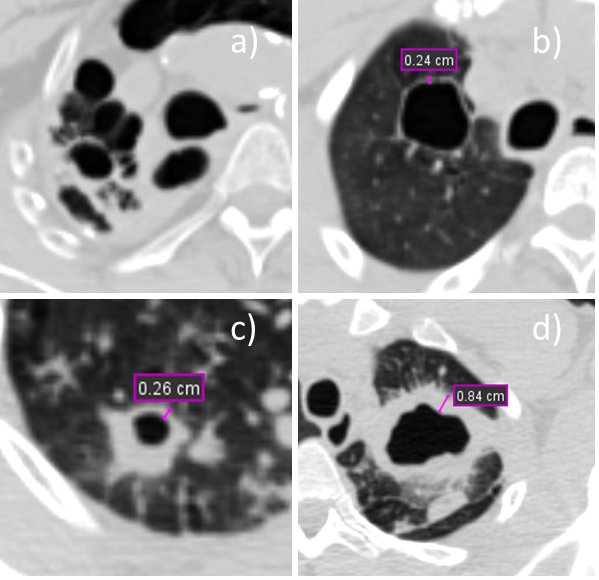


*Supplementary Figure 1 Examples of cavity lesions on transverse slices of M6 CT images.*

*a) A cured patient with extensive right upper lobe fibrotic changes and destruction. Cavity wall not enclosed, thus not measured.*

*b) Cavity with a 2.4 mm wall on the CT images of a cured patient.*

*c) A cured patient with 2.6 mm cavity and confluent nodule.*

*d) A failed treatment cases with a well-circumscribed cavity with an 8.4 mm wall.*

*.*

*
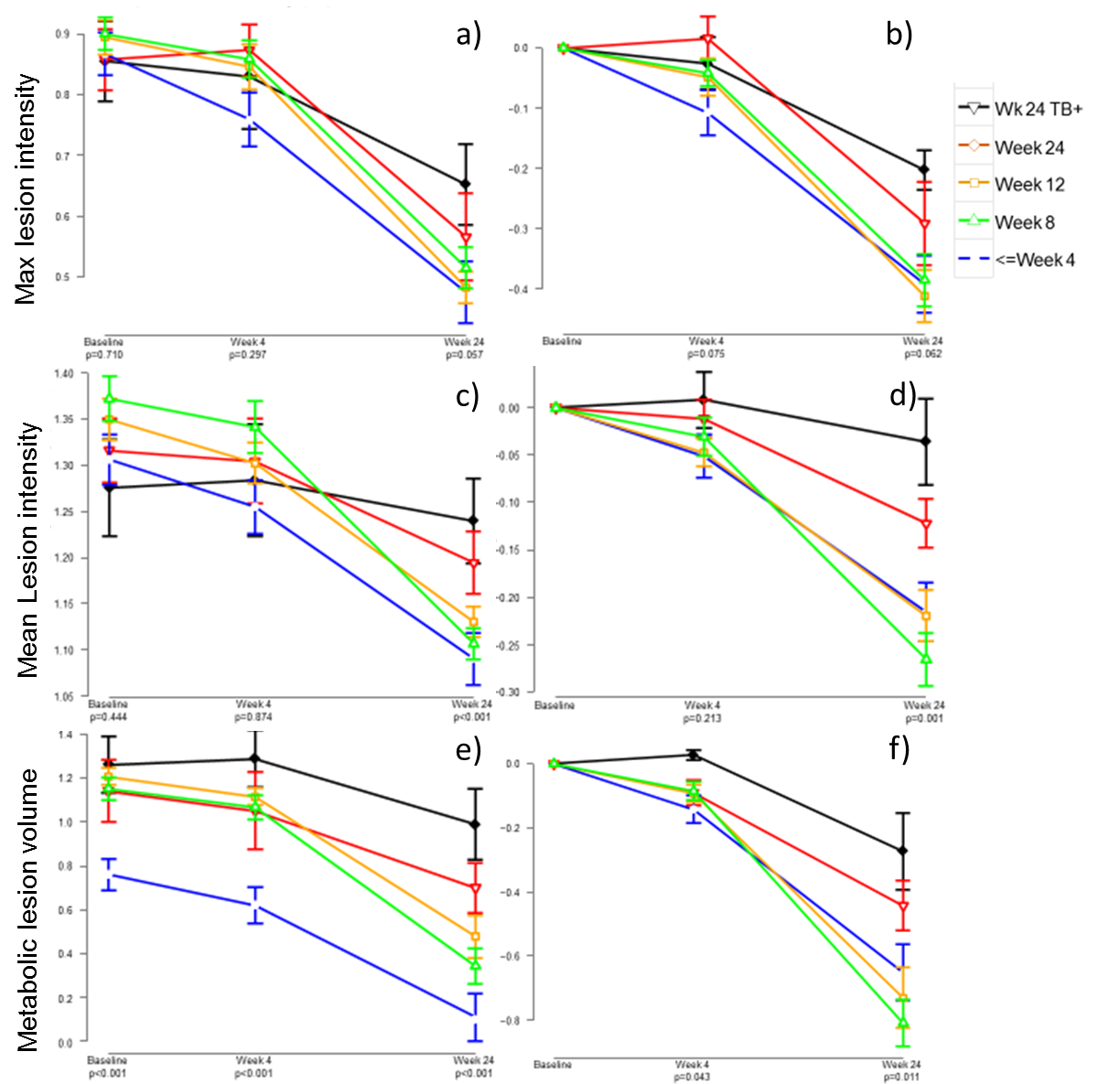
*

Supplementary figure 3

Mean (±SE) Log10 transformed values of PET parameters over time by time to negativity group. Maximum lesion intensity in a) SUVmax and b) change from baseline. Mean lesion intensity (Z_mean_) in c) mean Z-score and d) change from baseline. Metabolic lesion volume in e) percentage of total lung volume and f) change from baseline.

*
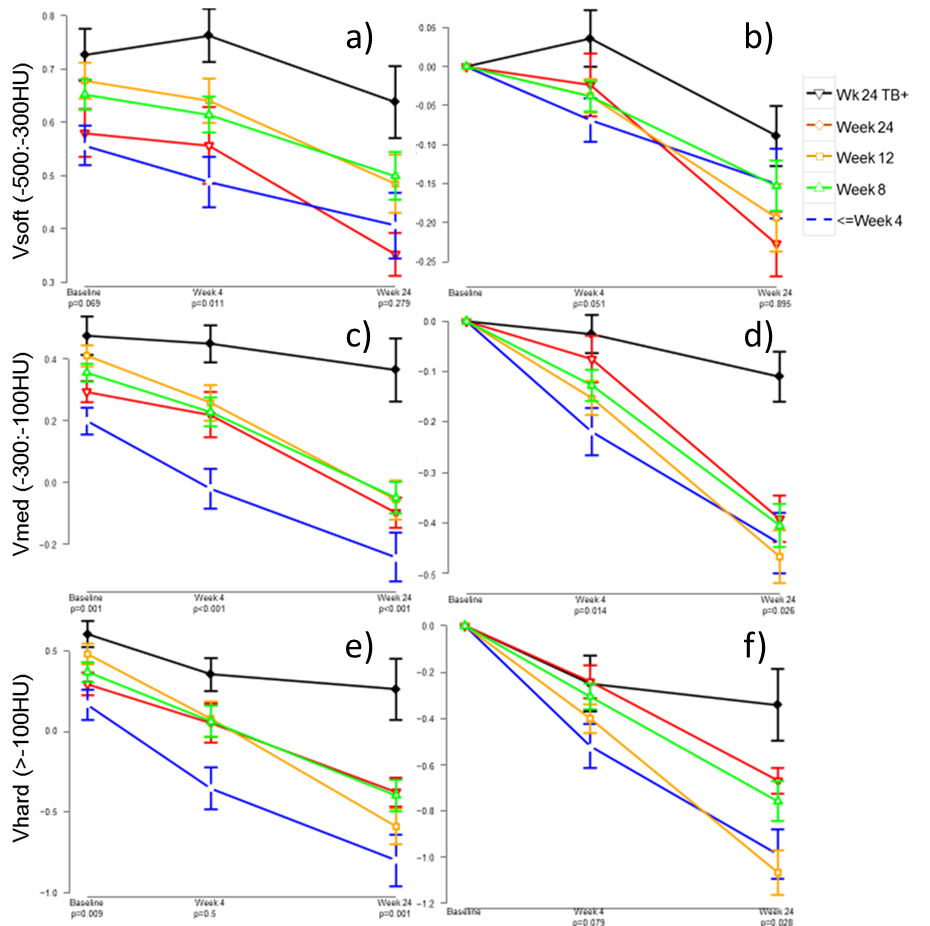
*

*Supplementary Figure 4*

Mean (±SE) Log10 transformed values of CT parameters at Dx, M1 and M6 by time to negativity group. Analysis of variance P-Values indicated. Soft lesions (V_soft_) in a) percentage of total lung volume and b) change from baseline. Medium lesions (V_medium_) in ac) percentage of total lung volume and d) change from baseline. Hard lesions (V_hard_) in e) percentage of total lung volume and f) change from baseline.

*
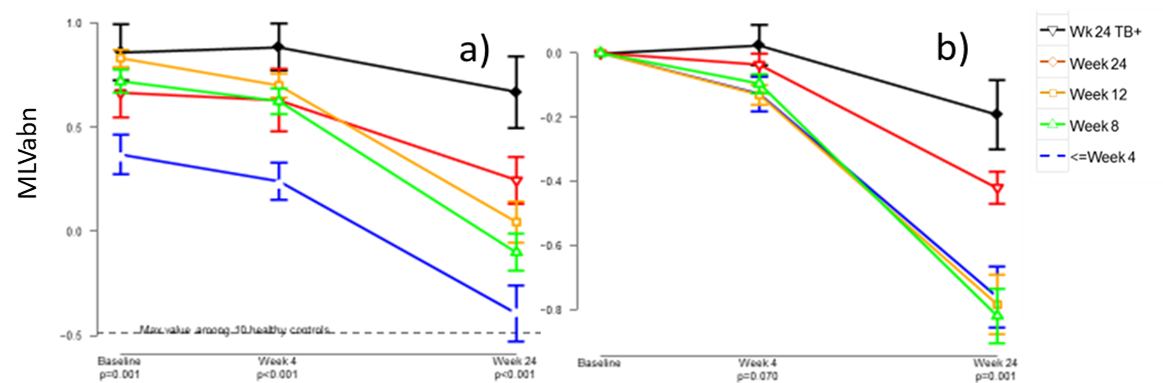
*

*Supplementary Figure 5*

Mean (±SE) Log10 transformed values of intersection of MLV and abnormal density at Dx, M1 and M6 by time to negativity group. Analysis of variance P-Values indicated. a) percentage of total lung volume and b) change from baseline.


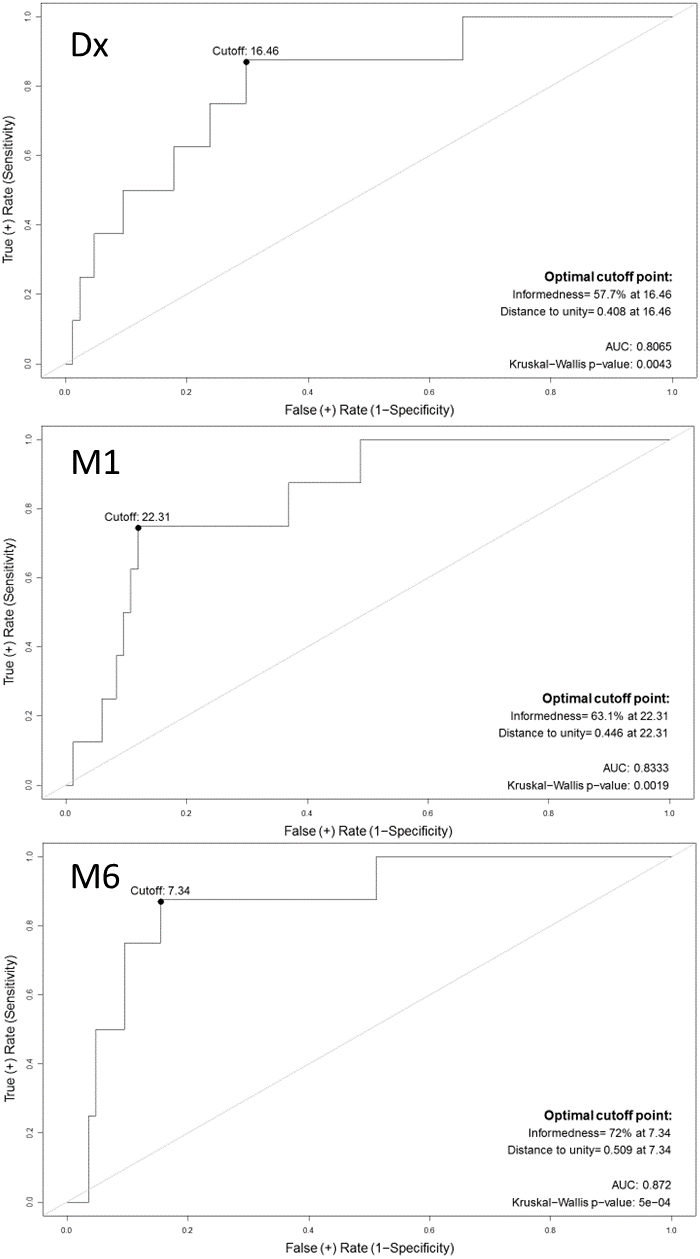


Supplementary figure 6 Receiver operating characteristic (ROC) curves of cavity volume predicting failed treatment.


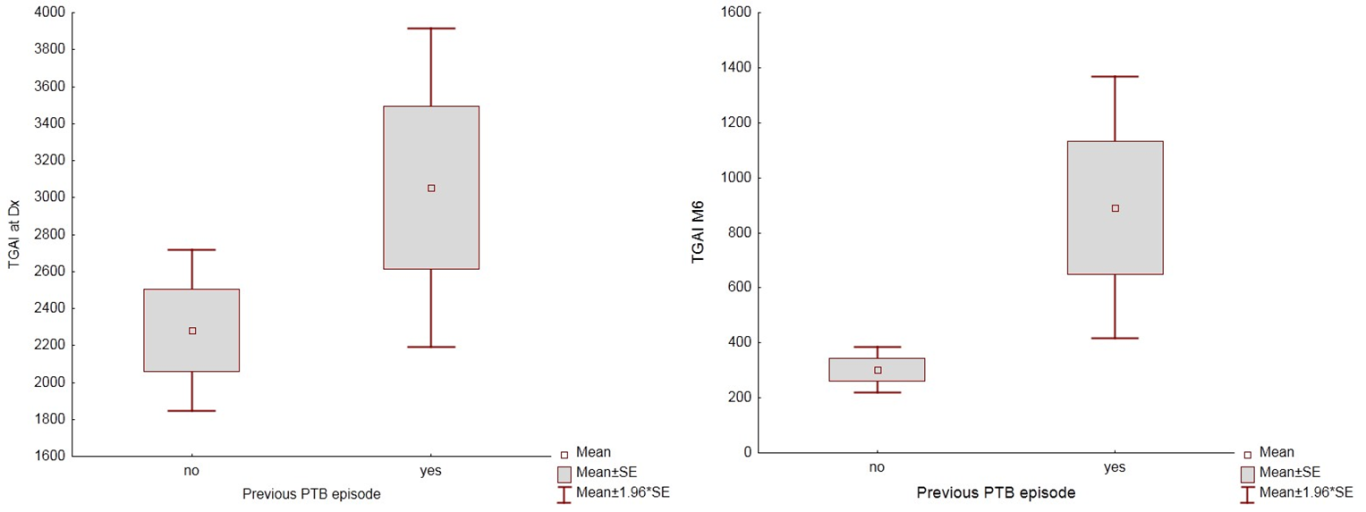


Supplementary Figure 7 Association with between a history of previous PTB episode(s) and TGAI at Dx and M6. Box and whisker plot showing Mean, ±SE, ±1.96×SE.

*
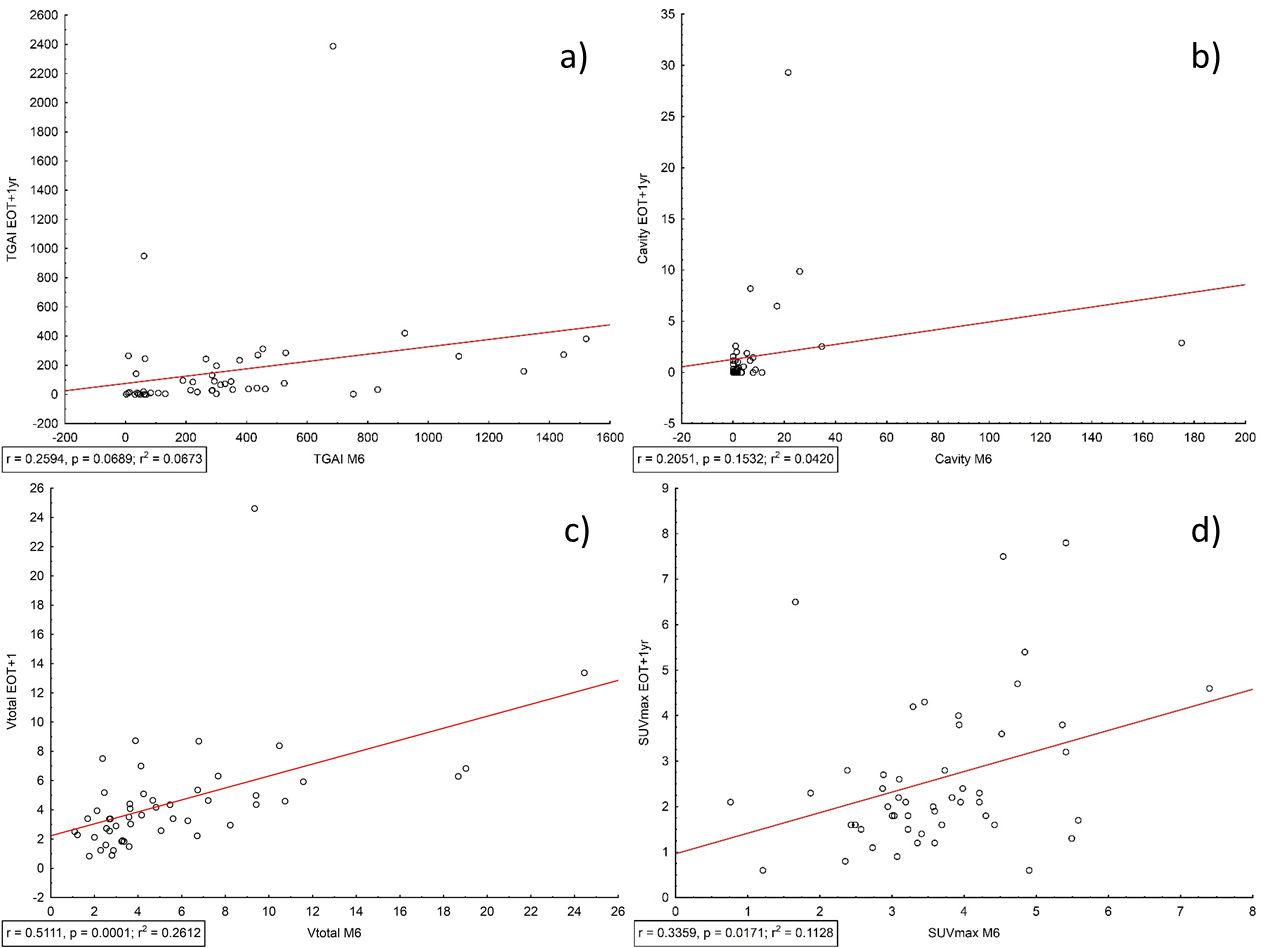
*

*Supplementary figure 8 Scatterplots to show correlation of PET/CT scan parameters at M6 and EOT + 1y.*

*a) TGAI, weak positive correlation, non-significant. b) Cavitation volume, no significant correlation. c) V_total_, significant positive correlation. d) SUVmax, moderate, but significant positive correlation.*
